# Carbon footprint of private dental laboratories in Egypt: A cross-sectional study

**DOI:** 10.1038/s41405-025-00316-w

**Published:** 2025-04-17

**Authors:** Amira H. Elwan, Ahmed Mahmoud Fouda

**Affiliations:** 1 Department of Pediatric Dentistry and Dental Public Health, Faculty of Dentistry, Alexandria University, Alexandria, Egypt; 2 Afrone Network, Faculty of Dentistry, Alexandria University, Alexandria, Egypt; 3 Department of Fixed Prosthodontics, Faculty of Dentistry, Suez Canal University, Ismailia, Egypt

**Keywords:** Prosthetic dentistry, Dental public health

## Abstract

**Background:**

Climate change poses a serious threat to the planet, mainly driven by greenhouse gas (GHG) emissions. Dental laboratories contribute to GHG emissions through staff travel, waste, energy and water consumption, and procurement. Carbon footprinting is the process of quantifying the direct and indirect GHG emissions associated with a service. This study aimed to assess the Carbon Footprint (CFP) of private dental laboratories in Egypt.

**Materials and methods:**

Data were collected from private dental laboratories in Cairo, Alexandria, and Elbeheira, Egypt in August 2024 through interview questionnaires. A CFP calculator was used to estimate carbon emissions from staff travel, waste, energy and water consumption, and procurement. The data of all laboratories was summed and divided to determine the average CFP per laboratory and per prothesis/appliance, both with and without the depreciation of dental equipment.

**Results:**

Data from 21 dental laboratories were collected. An average private dental laboratory in Egypt worked 309 days with a staff of around 7 persons and makes around 7119 prostheses/appliance per year. The CFP of dental laboratories was around 20,820 kg CO_2_e, equal to 2.9 kg CO_2_e per prosthesis/appliance. The largest contributor to the CFP was staff travel (43.6%), followed by procurement (27.8%), energy consumption (25%), waste (3.3%), and water consumption (0.1%). After including the depreciation of dental equipment, the CFP increased by 7.7%.

**Conclusion:**

Private dental laboratories in Egypt produce a significant amount of carbon emissions. Staff travel was the major contributor to the carbon emission because each laboratory hired several couriers to deliver the prostheses/appliances and impressions. The CFP of electricity consumption was significant, likely because the air conditioning ran throughout the year to cool the machines down. Future studies are needed to develop customized country-specific CFP calculators to accurately measure the carbon emissions of dental laboratories in various settings. Preventing oral diseases, educating technicians on sustainable dental practices, optimizing public transportation, using bulk delivery services, shifting to renewable energy, and adopting circular economy are essential to mitigate the carbon emissions of dental laboratories.

## Introduction

Dental laboratories, while essential for comprehensive treatment plans, have a considerable environmental impact. Many materials used in dental laboratories are single-use, such as impression trays and personal protective equipment, which contribute to plastic waste. Dental laboratories also utilize various chemicals, such as polymers, gypsum products, etchants, ceramics, metals, wax, and solvents, which can be harmful to the air, land, and water if not disposed of properly [1]. Dental laboratories rely on energy-intensive equipment like furnaces, ovens, and casting machines, which can result in high carbon emissions from energy consumption [2].

Fossil fuel combustion releases carbon dioxide in the atmosphere. Carbon dioxide is a potent greenhouse gas (GHG), which absorbs and traps heat within the atmosphere [3]. Global warming is the progressive gradual rise of the planet’s surface temperature that is caused by the GHG effect and results in changes in global climate patterns [4]. Climate change refers to a change in the state of the climate which can be identified by changes in the mean and/or the variability of its properties and persists for an extended period, such as decades or longer. Climate change could be due to natural internal processes or external forces [5]. Climate change has a disastrous impact on the planet, including increased heatwaves, droughts, floods, rising sea levels, and extreme weather events. The continuous monitoring of GHG emissions and low-carbon sustainable solutions are required to limit global warming to well below 2 °C [6].

Monitoring of GHG emissions is done through carbon footprinting, which quantifies the direct and indirect GHG emissions released from a service, product, or process. It combines all GHGs into one unit named carbon dioxide equivalent (CO_2_e) [7]. The main methodologies for carbon footprinting are Process-based Life Cycle Analysis (PB-LCA), Environmental Input-Output Life Cycle Analysis (EIO-LCA), and a hybrid method. PB-LCA estimates GHG emissions by tracking the pathways of supply chains and multiplying them by conversion factors derived from scientific studies. EIO-LCA, the spend-based approach, estimates GHG emissions by calculating the amount of money spent on a service and multiplying it by a sector-specific conversion factor [7].

The global healthcare sector produced 1.6 to 2 gigatons of CO_2_e in 2019, representing 4.4% of the world GHG emissions [8]. The GHG emissions of the global dental sector constitute around 3% of the global healthcare sector’s GHG emissions [9]. The GHG emissions of dental laboratories can be explained through The Greenhouse Gas Corporate Accounting and Reporting Standard Protocol [10]. It defines three broad scopes of GHG emissions (Table 1).

**Table 1:** Scopes of GHG emissions according to the Greenhouse Gas Protocol [10].

Scope		Route of carbon emissions
I	Direct GHG emissions	Emissions from the combustion of fuel in transport of dental products in vehicles owned or controlled by laboratories as well as natural gas combustion for heating
II	Indirect GHG emissions from energy production	Primarily emissions from the generation of electricity in power plants.
III	All other indirect emissions, including: 1→Staff travel	Emissions from the combustion of fuel in staff travel by vehicles not owned or controlled by laboratories. This also includes courier travel who deliver dental protheses from laboratories to clinics. Public transportation, for instance buses and trains, have less CFP per passenger-kilometer than cars. Cycling or walking have the lowest CFP [44]
	2→Waste	Consumption of energy for waste transportation from laboratories to incinerators or treatment facilities and the energy needed for incineration or deposition in a landfill. Incineration has a higher CFP than deposition in a landfill, while recycling has the least CFP [45].
	3→Water consumption	Consumption of energy for water acquisition, treatment, distributing, and sewage treatment
	4→Procurement	Consumption of energy for the extraction of raw materials, manufacturing, packaging, processing, distribution to laboratories [46]. Equipment constitutes a significant portion of spending in dental laboratories. Depreciation is the process of allocating the cost of equipment over its expected useful life, thereby reflecting the consumption of its value as it is used [47]. Thus, calculating the CFP of the periodic depreciation of dental equipment should be considered [28].

A systematic review showed that the CFP of laboratories is usually pooled with the CFP of other healthcare services [11]. The same approach was used to estimate the CFP of dental services in the National Health Service (NHS) England [2, 12–14]. A study showed that the laboratory fees constituted one third of the procurement fees of all NHS England dental clinics, accounting for 7679.3 kg CO_2_e [12]. However, this approach only accounted for the laboratory fees and may not fully reflect the CFP of dental laboratories [7, 12]. None of the previous studies solely assessed the CFP of dental laboratories.

Climate change disproportionately impacts the developing world, which has a limited capacity to adopt effective mitigation and adaptation strategies to reduce their projected vulnerability to climate change [15]. While Africa has negligibly contributed to climate change [16], it is one of the most vulnerable regions in the world. This is due to the low socioeconomic growth and high economic dependency on climate-related resources, such as fishing and agriculture [15]. To date, little is known about the CFP of oral healthcare in African and developing countries.

Egypt is a lower-middle income country located in North Africa [17]. Although Egypt shares by only 0.69% to the world’s CO_2_e emission [16], it is highly vulnerable to climate change. The Egyptian population growth (over 106 million people) is considered the major driver of escalating GHG emissions [17]. Climate projections indicate that Egypt will continue to experience a higher level of warming than the global average [18]. This necessitates the close monitoring of the CFP of healthcare facilities, especially the private healthcare sector, as it is the fastest-growing healthcare provider in Egypt [19]. This study aimed to assess the CFP of private dental laboratories in Egypt. This would help identify carbon-intensive areas, develop effective carbon mitigation strategies, enable targeted interventions for greener oral healthcare systems, and facilitate informed decision-making.

The Egyptian dental laboratory model, characterized by a mix of small laboratories and larger facilities, reliance on fossil fuel energy sources, and improper segregation of waste, may be representative of practices in other Low- and Middle-Income Countries LMICs. The study findings can be extrapolated to other LMICs, as they share similar socioeconomic contexts, including developing economies, limited resources, and nascent regulatory frameworks [20]. These shared characteristics often translate to similar challenges in energy production, transportation system, and waste management in the dental sector.

## Materials and methods

A cross-sectional study was conducted in Cairo, Alexandria and Elbeheira, Egypt in August 2024. Cairo, the capital and largest city of Egypt, has a population size of over 10 million people and an area of 3085 km². Alexandria, the second largest city of Egypt and its main port, has a population size of over 5 million people and a size of 2300 km². Elbeheira, a northwestern governorate in the Nile Delta, has a population size of around 7 million people and an area of 9826 km² [17]. There are no data on the number of private dental laboratories in Egypt. Ethical approval was obtained from the Research Ethics Committee, Faculty of Dentistry, Alexandria University, Egypt (#0941-07/2024). Informed consent was obtained from the laboratories’ managers, technicians, and personnel. Data were collected from private dental laboratories from different areas in the three governorates. A laboratory was considered eligible if it was a private standalone laboratory with at least one full-time technician. Public laboratories at hospitals were excluded. Laboratories within integrated healthcare facilities were ineligible due to the difficulty of assessing independent water and energy consumption.

Interview questionnaires were done, in person or over the phone, to collect year-round data from each laboratory about the workflow from July 2023 to July 2024. The validated Duane et al. CFP calculator for dental clinics [2] was used. The calculator consists of 8 items: (1) the number of days that the clinic is open per year, (2) the number of staff, (3) the number of patient visits per year, (4) staff travel distance and method of transportation, (5) number of waste bags (infectious, domestic, plastic waste for recycling, and cardboard waste for recycling), (6) Energy consumption (standard and green electricity, electricity from solar panels on clinic’s roof, and gas), (7) water consumption, (8) procurement (Expenditure on dental materials and equipment excluding rent and interest). The calculator was based on some assumptions (Table 2). The third question was replaced by the number of prostheses/appliances fabricated per year to calculate the CFP per prosthesis/appliance. The validity of the questionnaire was assessed by a panel of experts.

**Table 2:** The assumptions of the items used in the carbon footprint calculator [2]

Item	Assumptions
Staff travel	1→All staff traveled by petrol/diesel car*. 2→The size of cars was medium. 3→CFP of walking and cycling was zero.
Waste	1→Each waste bag weighed 6.72 kg. 2→CFP of recycling waste was zero. 3→Infectious waste was high-temperature incinerated.
Procurement	1→Emissions from procurement was assessed by the spend-based method. Every 1 Great Britain Pound (GBP) spent on dental materials and equipment was assumed to be equal to an average weighted conversion factor.

^*^Patients were assumed to travel by petrol/diesel car, electric car, bus, train, motorbike, walking, or cycling. The lower rate of car ownership in Egypt compared to England ( < 10% versus 78%, respectively) [48, 49] prompted us to investigate the types of vehicles that the staff used to commute to work then apply the appropriate emission factor for each vehicle type from the patient travel emission factors.

The annual depreciation of the equipment was calculated assuming that they will be replaced within 5 years [21]. The sources of data are shown in Table 3. Respondents, Laboratory managers and personnel were asked to provide year-round electricity, gas, and water bills and dental materials’ and equipment’s invoices. The data of all laboratories were averaged to obtain the average CFP per laboratory, per prosthesis/appliance, both with and without the depreciation cost. The carbon footprinting approach was PB-LCA, except for procurement which was EIO-LCA.

**Table 3:** The source of information for items used in the carbon footprint calculator.

Item	Source of information
Number of workdays per year	The laboratory manager was asked about the average number of days the laboratory was open per week and the number of days the laboratory was closed during national holidays.
Number of full-time staff	The number of full- and part-time technicians, receptionists, accountants, administrative personnels, cleaning and sterilization personnel, human resources personnel, and couriers were collected. Because the calculator uses the number of full-time staff for the calculation of staff travel per year, each two part-time staff were considered one full-time staff.
Number of prostheses/appliances that the laboratory makes per year (Indirect restorations and appliances, including crowns, bridges, veneers, removable dentures, surgical guides, and orthodontic and pedodontics appliances)	The laboratories did not keep record of the fabricated prostheses/appliances, so the laboratory manager was asked to record the number of prostheses/appliances fabricated in a week. This was averaged and multiplied by the average number of workdays in a year.
Staff travel to or for the laboratory by any of the following methods (petrol/diesel car, electric car, bus, train, motorbike, bike/walk) in miles	The staff were asked about their residence locations. The shortest distance from their residence to the laboratory, as calculated by the Global Positioning System (GPS), was used to determine staff travel distance for each transportation method and was multiplied by two to calculate the total distance per return journey. The distance that the part-time staff traveled was calculated and allocated according to the number of workdays. The total distance traveled by the staff was then summed for each transportation mode. The Tuk-Tuk (auto-rickshaw) is a three-wheeled transportation vehicle that is popular in less urbanized areas. It has the same engine design as the motorbike and emits equal carbon amounts [50]. The distance traveled by the Tuk-Tuk was calculated among the distance traveled by the motorbike. The tram and metro (underground) are popular electricity-powered mass transportation methods in Alexandria and Cairo, respectively. They emit more carbon per passenger-kilometer than electric cars, equivalent to a diesel train [44]. The distances traveled by the tram and metro were included in the calculations for train travel. Each laboratory hired several couriers, who traveled by motorbikes, to deliver the prostheses/appliances and dental impressions to and from the clinics. The laboratory manager was asked about the location of the clinics they deliver their services to by couriers and the frequency that the couriers made these rides per day. The average distance between the clinics and the laboratory was calculated and multiplied by the average number of rides per day to obtain the average distance traveled by each courier.
Number of waste bags that the laboratory discards per week*: Plastic waste for recycling, cardboard waste for recycling, infectious waste** for incineration and domestic waste for disposal (each bag weighing 6.72 kg)	The cleaning personnel were asked to count and weigh the waste bags discarded during an average week. This number was multiplied by the average weight of the bag and then divided by 6.72 kg [2].
Consumption of standard electricity, green electricity, solar power on the laboratory’s roof, and gas last year in kilowatt hour (kWh)	Energy consumption in last year’s bills was summed. If any monthly bill was missing, the average consumption was used as a substitute for that month. There were no gas bills because the laboratories used gas tanks instead of pipelines. The laboratory manager was asked about the average number of gas tanks used per month. This was multiplied by the average capacity of gas tanks in Egypt (7.93 gallons) and multiplied by a gasoline gas equivalent (36.6) to obtain the number of kWh consumed per month [51].
Water consumption last year in cubic meters (m^3^)	Water consumption in last year’s bills was summed. If any monthly bill was missing, the average consumption was used as a substitute for that month.
Expenditure on equipment and materials during the last year GBP (not including rent or interest)	Last year’s invoices for dental materials, equipment, and administrative costs were summed. The depreciation cost of equipment (porcelain, burnout and sintering furnaces, sandblasters, compressors, Computer-aided Design/ Computer-aided manufacturing (CAD/CAM) milling machines, computers, trimmers, Bunsen burners, extra-oral scanners, handpieces, micromotors, 3-D printer, trimmers, flasks, and washing and polishing machines, and induction casting machines) was calculated and added to the cost of procurement in a separate calculation. Procurement costs were converted to GBP by dividing the laboratories’ expenses by the average Egyptian Pound value during 2023-2024.

^*^This question assessed the waste management method, as the ultimate waste management method determines the resulting carbon emissions. Therefore, the accuracy of waste segregation on-site was not investigated [45].

**Infectious waste in dental laboratories mainly consists of impressions and models contaminated with blood and saliva [52]. This is particularly evident in Egypt. Studies showed that only 37.2% of the dental personnel disinfect impressions before sending it to the laboratory [53] and 71.4% of the technicians do not disinfect the received impressions [54].

## Results

Twenty-one dental laboratories (2 in Cairo, 16 in Alexandria, and 3 in Elbeheira) consented to participate in the study out of 24 invited (87.5%). The average number of workdays in the laboratories was 309 days. The average full-time staff number was around 7 persons. The average number of fabricated prostheses/appliances per year was 7,119.

The average total distance traveled by staff was 972.1 miles per week, accounting for 9083.6 kg CO_2_e per year. Motorbikes were the highest source of carbon emissions (5545.4), followed by buses (1754.1), and petrol/diesel cars (1680.6) kg CO_2_e. An average of 1.2 infectious waste bags and 3.9 domestic waste bags were collected per week. There were no waste bags for recycling. The total waste CFP was 697.1 kg CO_2_e per year.

The average yearly electricity and gas consumption was around 17,789 and 1540 kWh, which accounted for 4889.5 and 323.4 kg CO_2_e, respectively. No green electricity or solar power were consumed. The average yearly water usage was around 89 m^3^, accounting for 30.1 kg CO_2_e per year. The average procurement was around 44,088 GBP, accounting for 5786.1 kg CO_2_e per year.

**Fig. 1:**
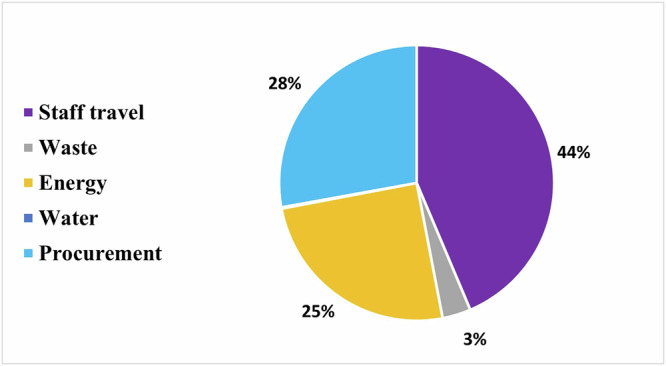
Distribution of the CFP of private dental laboratories in Egypt.

The average total CFP of a private dental laboratory in a year was around 20,820 kg CO_2_e, equal to 2.9 kg CO_2_e per prosthesis/appliance. This rose to around 22,426 Kg CO_2_e, 3.1 kg CO_2_e per prosthesis/appliance, after adding the cost of depreciation. Depreciation increased the CFP by 7.7% (Table 4). Staff travel represented 43.6% of the private dental laboratories CFP, followed by procurement (27.8%), energy consumption (25%), waste (3.3%) and water consumption (0.1%) (Fig. 1).

**Table 4:** The average annual carbon footprint of private prosthodontic dental laboratories in Egypt (*N* = 21).

Laboratory information		Average	Conversion factors	CFP per year Kg CO_2_e
Number of workdays per year		309.1	–	–
Number of full-time staff		6.6	–	–
Number of prostheses/ appliances per year		7119.4	–	–
Staff travel in a week (miles)	Petrol/diesel car	61.5	0.53	1680.6
	Electric car	0.0	0.18	0.0
	Bus	227.0	0.15	1754.1
	Train	10.6	0.19	103.5
	Motorbike	672.7	0.16	5545.4
	Bike/walk	0.3	0.00	0.0
	Total	972.1	–	9083.6
Number of waste bags in an average week	Plastic waste for recycling	0.0	0.00	0.0
	Cardboard for recycling	0.0	0.00	0.0
	Infectious waste for incineration	1.2	7.59	466.8
	Domestic waste for disposal	3.9	1.16	230.4
	Total	5.1	–	697.1
Energy consumption in a year (kWh)	Standard electricity	17,789.2	0.27	4889.5
	Green electricity	0.0	0.01	0.0
	Solar power	0.0	0.04	0.0
	Gas	1539.9	0.21	323.4
	Total	19,329.1	–	5212.9
Water consumption in a year (m^3^)		89.1	0.34	30.1
Procurement in a year (GBP)		44,087.9	0.13	5786.1
Procurement with depreciation in a year (GBP)		56,303.6	0.13	7402.1
CFP without depreciation in a year				20,819.8
CFP per prosthesis/ appliance without depreciation in a year				2.9
CFP with depreciation in a year				22,425.8
CFP per prosthesis with depreciation in a year				3.1

*CFP* Carbon footprint, *CO*_*2*_*e* Carbon dioxide equivalent, *km* kilometer, *kWh* kilowatt hour, *m*^*3*^ cubic meter, *GBP* Great British Pound.

## Discussion

The study findings showed that in private dental laboratories in Egypt, staff travel was the greatest contributing factor to the CFP, followed by procurement and energy consumption, whereas waste and water had less impact.

Private dental laboratories in Egypt are not bound to a fixed work schedule, which contributes to the notable number of workdays. Additionally, the number of full-time staff in private laboratories could be attributed to the low salaries in Egypt [22], which allowed the laboratories to hire a significant number of staff. High workdays and staff number contributed to a high CFP of staff travel. The CFP calculator was designed according to travel in England, however, public transportation in Egypt may be older and less efficient [23], so we might be underestimating the CFP for staff travel in Egypt. It is important to invest in improving roads and public transportation for optimal fuel efficiency and to encourage energy-efficient mass transportation, such as electric buses and trams. Notably, the majority of staff travel CFP was attributed to motorbike travel, because the laboratories hired multiple couriers to deliver the prostheses/appliances and dental impressions to and from the clinics. Thus, dental laboratories could benefit from bulk delivery services which deliver multiple packages to different addresses while saving time and fuel.

The remarkable CFP of procurement could be attributed to the high number and diversity of dental materials needed for the fabrication of prostheses/appliances. However, the carbon calculator used the spend-based approach to estimate the CFP of procurement. The conversion factor was based on the carbon emissions of the NHS expenses, adjusted for the inflation in England [14]. This generic conversion factor may not accurately reflect the procurement CFP in other countries, as in Egypt, because both countries have different oral healthcare expenditure, inflation rates and supply chains. The NHS allocates considerably more annual funding to oral health care than Egypt (3 billion and 0.257 ≈ 0.3 billion GBP, respectively) [24, 25]. The inflation rate, in the present time, is 2.2% in England and 25.7% in Egypt [26]. Egypt imports a considerable amount of its dental products from European countries, which denotes longer transportation distances and supply chains [27], thus higher CFP. These differences in the CFP components for procurement between both countries emphasize the need to develop customized country-specific calculators to precisely assess the CFP of dental laboratories in different settings. Additionally, there is a need to locally manufacture dental machinery and equipment, thereby shorten the supply chain and eliminate the carbon emissions associated with cross-country transport.

Calculating the CFP of dental laboratories taking into consideration the depreciation of existing equipment resulted in a distinct increase in the CFP of procurement. This highlights the importance of investing in equipment with prolonged durable life, because such equipment decreases the need for frequent replacements, saving costs associated with purchasing, transporting, and installing new equipment. This ultimately contributes to improved environmental efficiency and more sustainable value chains [28].

There was a notable CFP of electricity consumption. This could be explained by several factors. First, the high temperature and humidity necessitated running the air conditioning at low temperatures [29]. Second, dental laboratories employ electricity-intensive machines, such as the CAD/CAM machines and furnaces [30]. Third, the air conditioning was often used year-round to cool the machines down. Fourth, the shortage of technicians led to extended work hours to meet the high demand of the dental clinics [31], resulting in prolonged electricity consumption. Conversely, gas consumption was minimal, as it was primarily used for waxing and was not needed during winter for heating [29]. Neither green electricity nor solar panels were used. Egypt utilizes electricity produced from a fuel mix, mainly fossil fuel (88%) [32]. This results in a higher CFP of electricity consumption. Transitioning to renewable energy is advised to mitigate carbon emissions. Dental laboratories’ buildings should be designed with thermal efficiency in mind to minimize the use of air conditioning and its associated GHG emissions [33].

The noticeable CFP of waste could be attributed to the lack of training that the technicians receive on waste segregation [34], lack of recycling, and deficient incineration facilities [35]. Reusing dental instruments, such as impression trays and finishing and polishing burs, after thorough cleaning, disinfection, and sterilization could help reduce waste CFP [36]. Recycling dental materials used in the laboratories, such as gypsum, acrylic resin, and orthodontic wires, could decrease the demand for raw materials and minimize waste. Rethinking the entire supply chain is needed to shift from the linear to circular economy models [37]. Linear economy is characterized by the consumption and disposal of products, leading to resources depletion, increased procurement rates, and end-of-life waste. Instead, circular economy involves reducing, reusing, and recycling resources throughout production, distribution, and consumption processes, thus, decreasing the CFP of waste, procurement and energy consumption along the entire supply chain [38].

The CFP of water consumption was not significant because water was only needed for mixing of gypsum and domestic purposes. In addition, some laboratories relied on digital fabrication of prostheses which involved less water consumption. The estimated CFP per prosthesis/appliance highlights the substantial environmental impact of oral diseases complications and tooth loss, in addition to their well-documented effects on health, community, and economy [39]. This underscores the necessity of considering the environmental impact as a part of the overall burden of oral diseases. There should be a collaborative effort among dental clinicians, laboratories, and regulatory bodies to rationalize the use of indirect restorations while considering both carbon emissions and patient comfort and satisfaction.

This study has several strengths. First, the study sheds light on the CFP of dental laboratories solely, unlike other studies which pooled the CFP of laboratories with dental clinics and hospitals [2, 12–14]. Second, the carbon footprinting of staff travel considered country-specific transportation methods, such as the Tuk-Tuk, which had lower carbon emissions than other vehicles. Third, considering the courier services used by the laboratories revealed a distinct factor which largely increased the CFP of Egyptian dental laboratories, highlighting its associated challenges. Fourth, the calculation of the depreciation of dental equipment identified a previously overlooked source of GHG emissions that accumulates over the equipment’s useful life.

Regardless, this study has some limitations. First, the CFP calculator was designed according to the dental practice in England, which may not accurately reflect carbon emissions in Egyptian dental laboratories and suggests the need to develop customized country-specific CFP calculators. Second, the data may not have been measured with absolute precision due to the complexities and uncertainties of the estimation of dental products’ supply chains, waste weighing, couriers travel, and gas consumption [14]. Third, the laboratories did not maintain records of the number of prostheses/appliances produced, so the measured CFP per prosthesis/appliance may not be accurate. Fourth, as the CFP estimation is typically limited to a specific timeframe (one year) [10], it did not account for carbon emissions resulting from the construction of dental laboratories, as none were recently built.

The study findings can be extrapolated beyond Egypt, particularly for other LMICs facing similar challenges in the dental sector. For example, the staff travel CFP underscores the need for exploring more sustainable energy solutions in countries with heavy reliance on fossil fuels [40]. Similarly, the waste CFP highlights a common issue in LMICs countries where recycling infrastructure may be limited [41]. However, the diversity of cultural practices across LMICs necessitates a cautious approach to extrapolating the study findings [20]. Future research should investigate the specific contexts of other LMICs to allow for comparison and develop tailored solutions.

It is recommended that dental laboratories foster environmental sustainability by implementing digital dentistry technology, e.g. CAD-CAM and 3D printing of dental restorations. Digital dentistry promotes higher precision and accuracy over conventional dentistry. This contributes to shorter laboratory work hours, less energy and water use, less frequent need for remaking restorations, thus less staff travel. Digital dentistry eliminates the need for dental impressions, gypsum products, and waxes which considerably lowers waste and procurement [42]. Further research is needed to compare the environmental impact of conventional and digital dentistry in dental laboratories.

As demand for oral healthcare increases, the corresponding carbon emissions will likely intensify, if oral diseases are not sustainably managed. Preventing oral diseases remains the most effective strategy for reducing carbon emissions of dental laboratories, as it reduces oral diseases, disease complications, and the need for indirect restorations [43]. The regulating bodies should increase the monitoring and supervision of dental laboratories. Continuous education courses should be conducted to educate dental technicians on carbon reduction interventions and to facilitate the adoption of sustainable practices.

## Conclusion

Private dental laboratories in Egypt produce a substantial amount of carbon emissions. Staff travel was the primary contributor to these emissions mostly because laboratories hired multiple couriers to deliver the prostheses/appliances and impressions to and from the clinics. The CFP of electricity consumption was significant as the dental laboratories tended to keep the air conditioning on to cool down energy-intensive machines year-round. However, the CFP of gas consumption was minimal as heating was rarely needed. The CFP of procurement was substantial, highlighting the need to adopt a circular economy and local manufacturing of dental equipment. The notable CFP of waste was probably due to the lack of recycling and segregation. Future studies are needed to develop country-specific CFP calculators to accurately measure the carbon emissions of dental laboratories in various settings. Preventing oral diseases, regular monitoring of dental laboratories, educating technicians on sustainable dental practices, optimizing public transportation, shifting to renewable energy sources, and implementation of recycling are recommended to reduce carbon emissions of dental laboratories.

### Note added to proof

Note that elements of this study, particularly the methods, have been published as part of a related study in a different setting which was published during the production of this paper [55].

## Data Availability

The dataset used and/or analyzed during the present study is available from the corresponding author on reasonable request.

## References

[1] Shinkai RSA, Biazevic MGH, Michel-Crosato E, de Campos TT. Environmental sustainability related to dental materials and procedures in prosthodontics: A critical review. J Prosthetic Dent. 2023:S0022-3913(23)00370-0. 10.1016/j.prosdent.2023.05.024. Epub ahead of print. 10.1016/j.prosdent.2023.05.024 37709614

[2] Duane B, Steinbach I, Mackenzie L. A carbon calculator: the development of a user-friendly greenhouse gas measuring tool for general dental practice (Part 2). Br Dent J 2024;236:57–61. 38225322 10.1038/s41415-023-6626-7

[3] Lashof DA, Ahuja DR. Relative contributions of greenhouse gas emissions to global warming. Nature. 1990;344:529–31.

[4] United Nations. United Nations Framework Convention on Climate Change (UNFCCC) 1992. Available from: https://unfccc.int/resource/docs/convkp/conveng.pdf. Accessed 6 Sep 2024. 10.1111/resp.14705 38499332

[5] Intergovernmental Panel on Climate Change (IPCC). Global Warming of 1.5 °C. An IPCC Special Report on the impacts of global warming of 1.5 °C above pre-industrial levels and related global greenhouse gas emission pathways 2018 [666]. Available from: https://www.ipcc.ch/sr15/. Accessed 6 Sep 2024.

[6] The United Nations Framework Convention on Climate Change. Paris Climate Change Conference 2015. Available from: https://unfccc.int/process-and-meetings/the-paris-agreement. Accessed 5 Sep 2024.

[7] Pandey D, Agrawal M, Pandey JS. Carbon footprint: current methods of estimation. Environ Monit Assess 2011;178:135–60. 20848311 10.1007/s10661-010-1678-y

[8] Karliner J, Slotterback S, Boyd R, Ashby B, Steele K, Wang J. Health care’s climate footprint: the health sector contribution and opportunities for action. Eur J Public Health. 2020;30:ckaa165.843.

[9] Eckelman MJ, Sherman J. Environmental impacts of the U.S. Health Care system and effects on public health. PLoS ONE 2016;11:e0157014. 27280706 10.1371/journal.pone.0157014 PMC4900601

[10] World Business Council for Sustainable Development, World Resources Institute. The greenhouse gas protocol: a corporate accounting and reporting standard. Geneva, Switzerland, Washington, DC: World Business Council for Sustainable Development; World Resources Institute; 2004.

[11] Alshqaqeeq F, Amin Esmaeili M, Overcash M, Twomey J. Quantifying hospital services by carbon footprint: A systematic literature review of patient care alternatives. Resour Conserv Recycl 2020;154:104560.

[12] Public Health England Center for Sustainable Development. Carbon modelling within dentistry towards a sustainable future 2018. Available from: https://www.gov.uk/government/publications/carbon-modelling-within-dentistry-towards-a-sustainable-future. Accessed 05 July 2024.

[13] Duane B, Lee MB, White S, Stancliffe R, Steinbach I. An estimated carbon footprint of NHS primary dental care within England. How can dentistry be more environmentally sustainable? Br Dent J 2017;223:589–93. 29074898 10.1038/sj.bdj.2017.839

[14] Duane B, Steinbach I What is the environmental footprint of a dental practice? A life cycle analysis (Part 1). British Dental Journal. 2024. 10.1038/s41415-023-6710-z 38212528

[15] Ravindranath NH, Sathaye JA Climate Change and Developing Countries. In: Ravindranath NH, Sathaye JA, editors. Climate Change and Developing Countries. Dordrecht: Springer Netherlands; 2002. p. 247-65.

[16] Worldometer. CO2 Emissions by Country 2024. Available from: https://www.worldometers.info/co2-emissions/co2-emissions-by-country/. Accessed 19 Dec 2024.

[17] State Information Service. Local Administration in Egypt 2024. Available from: https://www.sis.gov.eg/section/2565/16?lang=en-us. Accessed 19 Dec 2024.

[18] Climate Change Knowledge Portal. Climate Data Projections - Egypt 2023. Available from: https://climateknowledgeportal.worldbank.org/country/egypt/climate-data-projections. Accessed 2 Sep 2024.

[19] Rashad AS, Sharaf MF. Who benefits from public healthcare subsidies in Egypt? Soc Sci 2015;4:1162–76.

[20] World Bank Group. Low and middle income countries 2025. Available from: https://data.worldbank.org/country/low-and-middle-income. Accessed 07 Feb 2025.

[21] Calculator.net. Depreciation Calculator 2024. Available from: https://www.calculator.net/depreciation-calculator.html. Accessed 11 Dec 2024.

[22] Kabbash I, El-Sallamy R, Zayed H, Alkhyate I, Omar A, Abdo S. The brain drain: why medical students and young physicians want to leave Egypt. East Mediterr Health J 2021;27:1102–8. 34927714 10.26719/emhj.21.050

[23] Ramadan I, El Toukhy M, Hussien KZ, Tosti F, Shaaban IG. Effect of Road, Environment, Driver, and Traffic Characteristics on Vehicle Emissions in Egypt. Int J Civ Eng 2022;20:1261–76.

[24] British Dental Assosiation (BDA). A billion in cuts 2024. Available from: https://www.bda.org/news-and-opinion/news/a-billion-in-cuts/. Accessed 4 Sep 2024.

[25] World Health Organization (WHO). Oral Health Egypt 2022 country profile 2022. Available from: https://www.who.int/publications/m/item/oral-health-egy-2022-country-profile. Accessed 2 Sep 2024.

[26] Trading Economics. Inflation rate in the world 2024. Available from: https://tradingeconomics.com/country-list/inflation-rate?continent=world. Accessed 5 Sep 2024.

[27] The Observatory of Economic Complexity. Dental Products in Egypt 2022. Available from: https://oec.world/en/profile/bilateral-product/dental-products/reporter/egy. Accessed 2 Sep 2024.

[28] Baxter J, Callewaert P, Danielsen R. Accounting the effects of product reuse and repair in life-cycle assessment. Clean Eng Technol 2024;21:100774.

[29] William MA, El-Haridi AM, Hanafy AA, El-Sayed AE-HA. Assessing the Energy Efficiency improvement for hospitals in Egypt using building simulation modeling. ERJ Eng Res J 2019;42:21–34.

[30] Triebe MJ, Mendis GP, Zhao F, Sutherland JW. Understanding energy consumption in a machine tool through energy mapping. Procedia Cirp 2018;69:259–64.

[31] Mabrouk MS, Marzouk SY, Afify HM. Investigation of quality improvement strategies within Egyptian dental clinics. Biomed Eng Appl Basis Commun 2019;31:1950006.

[32] International Energy Agency. Egypt’s Energy Supply 2025. Available from: https://www.iea.org/countries/egypt/energy-mix. Accessed 07 Feb 2025.

[33] El Shihy AA, Adel O, AlShanwany H. Thermal comfort evaluation in egyptian residential buildings: a case study to determine the problems of excessive electricity consumption. Egypt Int J Eng Sci Technol 2023;42:29–37.

[34] Haralur SB, Al-Qahtani AS, Al-Qarni MM, Al-Homrany RM, Aboalkhair AE, Madalakote SS. The dental solid waste management in different categories of dental laboratories in Abha City, Saudi Arabia. Open Dent J 2015;9:449–54. 26962373 10.2174/1874210601509010449 PMC4768654

[35] Milik SM. Assessment of solid waste management in Egypt during the last decade in light of the partnership between the Egyptian government and the private sector. Cairo, Egypt: American University in Cairo, AUC Knowledge Fountain; 2021. Available from: https://fount.aucegypt.edu/retro_etds/2473. Accessed 2 Feb 2025.

[36] Unger SR, Landis AE. Comparative life cycle assessment of reused versus disposable dental burs. Int J Life Cycle Assess 2014;19:1623–31.

[37] Martin N, Sheppard M, Gorasia G, Arora P, Cooper M, Mulligan S. Drivers, opportunities and best practice for sustainability in dentistry: A scoping review. J Dent 2021;112:103737. 34182061 10.1016/j.jdent.2021.103737

[38] Kirchherr J, Reike D, Hekkert M. Conceptualizing the circular economy: An analysis of 114 definitions. Resour Conserv Recycl 2017;127:221–32.

[39] Kassebaum NJ, Bernabé E, Dahiya M, Bhandari B, Murray CJL, Marcenes W. Global burden of severe tooth loss:a systematic review and meta-analysis. J Dent Res 2014;93:20S–8S. 24947899 10.1177/0022034514537828 PMC4293725

[40] Fuchs JL, Tesfamichael M, Clube R, Tomei J. How does energy modelling influence policymaking? Insights from low- and middle-income countries. Renew Sustain Energy Rev 2024;203:114726.

[41] Vinti G, Vaccari M. Solid Waste Management in Rural Communities of Developing Countries: An Overview of Challenges and Opportunities. Clean Technol 2022;4:1138–51.

[42] Duane B, Harford S, Steinbach I, Stancliffe R, Swan J, Lomax R, et al. Environmentally sustainable dentistry: energy use within the dental practice. Br Dent J 2019;226:367–73. 30850795 10.1038/s41415-019-0044-x

[43] Hyde S, Dupuis V, Mariri BP, Dartevelle S. Prevention of tooth loss and dental pain for reducing the global burden of oral diseases. Int Dent J 2017;67:19–25. 29023745 10.1111/idj.12328 PMC9378922

[44] Our World in Data. Which form of transport has the smallest carbon footprint? 2023. Available from: https://ourworldindata.org/travel-carbon-footprint. Accessed 2 Sep 2024.

[45] Unegg MC, Steininger KW, Ramsauer C, Rivera-Aguilar M. Assessing the environmental impact of waste management: A comparative study of CO2 emissions with a focus on recycling and incineration. J Clean Prod 2023;415:137745.

[46] Batsford H, Shah S, Wilson GJ. A changing climate and the dental profession. Br Dent J 2022;232:603–6. 35562450 10.1038/s41415-022-4202-1 PMC9100308

[47] Bierman H. A further study of depreciation. Account Rev 1966;41:271–4.

[48] Statista. Number of licensed vehicles in Egypt from 2022, by type 2022. Available from: https://www.statista.com/statistics/1378979/egypt-number-licensed-vehicles-by-type/#:~:text=In%202022%2C%20over%205.11%20million,2022%20reached%209.94%20million%20units. Accessed 2 Sep 2024.

[49] Department for Transport. National Travel Survey 2022: Household car availability and trends in car trips 2023. Available from: https://www.gov.uk/government/statistics/national-travel-survey-2022/national-travel-survey-2022-household-car-availability-and-trends-in-car-trips. Accessed 2 Sep 2024.

[50] The International Council on Clean Transportation. Air emissions from two- and three-wheelers: Initial issues assessment 2007. Available from: https://theicct.org/publication/air-emissions-from-two-and-three-wheelers-initial-issues-assessment/. Accessed 3 Sep 2024.

[51] Conversion of Measurement Units. Convert gallon to kWh 2024. Available from: https://www.convertunits.com/from/gallon/to/kWh#google_vignette. Accessed 24 Sep 2024.

[52] Mitsika I, Chanioti M, Antoniadou M. Dental solid waste analysis: a scoping review and research model proposal. Appl Sci 2024;14:2026.

[53] Gawish A, Khalifa A. Infection control practices among group of dental health care providers. Egypt Dent J 2016;62:971–6.

[54] Elkholy S, Sedky N. Application of infection control procedures in dental laboratories in Alexandria governorate and the efficacy of various disinfectants on the mostly used impression materials. Egypt Dental J. 2012;58:2377–87.

[55] Elwan AH, Tantawi ME, Fouda AM. Carbon footprint of private dental clinics in Egypt: a cross-sectional study. BMC Oral Health 2025;25:93. 10.1186/s12903-024-05413-0 10.1186/s12903-024-05413-0 PMC11742210 39825329

